# Supplementary material for: Diagnosis of Trypanosoma cruzi infection in Mexican populations: current conventional serology lacks adequate sensitivity and specificity
Source: Mem Inst Oswaldo Cruz. 2025 Oct 20;120:e240224. doi: 10.1590/0074-02760240224 (PMC12543361; doi:10.1590/0074-02760240224)
Supplement: Supplementary file 1 [file 1678-8060-mioc-120-e240224-s.pdf]

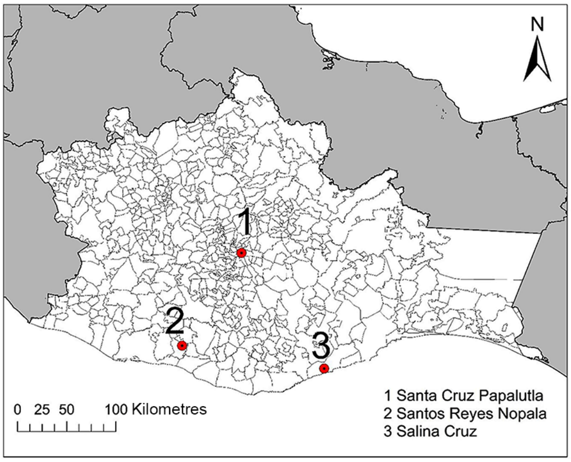

Fig. 1: site locations of Santos Reyes Nopala (SRN), Santa Cruz Papalutla (SCP) and Salina Cruz (SC) in Oaxaca, Mexico.

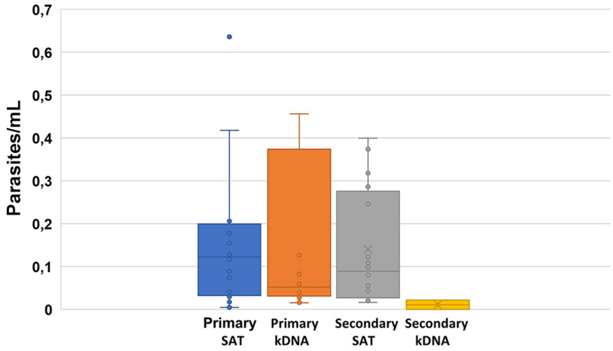

Fig. 2: adjusted parasite loads for quantifiable samples from TaqMan quantitative polymerase chain reaction (qPCR) using satellite DNA (SAT) for primary diagnosis group (PDG, N = 26) and secondary diagnosis group (SDG, N = 16) and kinetoplast DNA (kDNA) (PDG N = 23; SDG N = 6). Plot internal line represents median values included in Table III. High parasite load outliers for Santa Cruz Papalutla/PDG using SAT (1488, 2 parasites eq/mL) and kDNA (5, 8, 10 para eq/mL) are not represented.

TABLE I

Demographic and *Trypanosoma cruzi* infection exposure variables for study participants having previous (secondary diagnosis group - SDG) or no previous (primary diagnosis group - PDG) infection diagnosis, from three Oaxaca counties: Santos Reyes Nopala (SRN), Santa Cruz Papalutla (SCP) and Salina Cruz (SC)

	County (N)							
	Salina Cruz (SC, 17)		Santos Reyes Nopala (SRN, 27)		Santa Cruz Papalutla (SCP, 37)		Oaxaca (81)	
Elevation masl (region)	20 (Isthmus coast)		460-1200 (So. Sierra Madre)		1580 (Central Valleys)			
Population	76,450		4,580-1,380		1,730			
Vector species	*T. phyllosoma*		*T. mazzottii*, *T. dimidiata* Hg2		*T. barberi*			
T. cruzi diagnosis (N)	PDG (13)	SDG (4)	PDG (12)	SDG (15)	PDG (19)	SDG (18)	PDG (44)	SDG (37)
Ratio women/men	1.6	1.0	1.0	14.0	1.7	1.6	1.4	2.7
Mean age	37.4	42.3	49.1	68.6	40.9	51.7	42.0	57.5
Age range	13-65	25-65	26-79	40-96	15-72	16-76	13-79	16-96
Average schooling (yr)	8	9	7	1	7	5	7	4
Correct vector identification	75.0%	100.0%	77.8%	100.0%	31.6%	50.0%	62.5%	71.0%
Bugs observed in house	90.0%	100.0%	33.3%	46.2%	42.1%	66.7%	51.2%	68.6%
Bitten by bugs	90.9%	100.0%	57.1%	50.0%	22.2%	33.3%	42.9%	40.0%
Time since contact	3 yrs	> 10 yrs	5 yrs	> 10 yrs	5 yrs	> 10 yrs	3-10 yrs	> 10 yrs
Occurrence chinchoma	100.0%	100.0%	60.0%	54.5%	29.4%	11.1%	43.9%	32.4%
Chinchoma mean size (range)	4 (2-9)	5 (2-7)	4 (1-8)	3 (1-8)	2 (1-4)	1 (1-3)	3 (1-9)	3 (1-8)
Chinchoma duration (d)	5	3	5	3	6	4	6	4
Severity itching	average	severe	severe	severe	severe	severe	severe	severe
Animals in domicile	63.6%	100.0%	83.3%	92.3%	57.9%	94.4%	66.7%	91.2%
Fowl	54.5%	50.0%	83.3%	92.3%	47.4%	83.3%	59.5%	82.4%
Livestock	45.5%	50.0%	25.0%	30.8%	52.6%	72.2%	40.5%	52.9%
Pets	90.9%	100.0%	83.3%	76.9%	73.7%	100.0%	76.2%	91.2%
Pets visit field	45.5%	0.0%	70.0%	69.2%	63.2%	66.7%	57.1%	61.8%
Family member sleeps outside	36.4%	0.0%	66.7%	69.2%	5.3%	16.7%	31.0%	35.3%
Blood transfusion	18.2%	25.0%	25.0%	7.7%	21.1%	0 (18)	21.4%	2.9%
SSA	36.4%	75.0%	50.0%	53.8%	78.9%	88.9%	59.5%	76.5%
IMSS/IMSS Prospera	27.3%	0.0%	16.7%	30.8%	10.5%	5.6%	16.7%	14.7%
ISSSTE	27.3%	0.0%	25.0%	15.4%	0.0%	0.0%	14.3%	5.9%
Pemex	9.1%	0.0%	0.0%	0.0%	0.0%	0.0%	2.4%	0.0%

masl: metres above sea level; SSA: Secretaria de Salud y Asistencia; IMSS: Instituto Mexicano de Servicio Social; IMSS Prospera: Prospera program of IMSS; ISSSTE: Instituto de Seguridad y Servicios Sociales de los Trabajadores del Estado - Federal government worker healthcare program; Pemex: Health services of Petroleos Mexicanos.

TABLE II

Trypanosoma cruzi detection thresholds (based on sequence from expected-size bands) for five gene fragments using end point polymerase chain reaction (epPCR) and sensitivity of each gene to amplify 57 parasite populations either in addition to other genes (overall) or alone.

		T. cruzi infections identified		
Fragment	Detection threshold epPCR (para/mL)	Overall (57)	Alone (33)	Using > 1 gene (24)
SAT	5×10^{-3}	94.7%	30 (55.6%)	24 (44.4%)
kDNA	5×10^{-2}	26.3%	1 (6.7%)	14 (93.3%)
18S	5	8.8%	0	5 (100.0%)
ME	5×10^{2}	14.0%	2 (25.0%)	6 (75.0%)
24S	5×10^{3}	0.0%	0	-

SAT: satellite DNA; kDNA: kinetoplast DNA; 18S: small subunit 18S ribosomal; ME: mini-exon; 24S: 24Sα ribosomal.

TABLE III

Trypanosoma cruzi specificity of sequences from expected-size bands of five gene fragments amplified from Oaxaca populations

	SRN		SCP		SC		Oaxaca	
Fragment	NA	*T. cruzi* of amplified (N)	NA	*T. cruzi* of amplified (N)	NA	*T. cruzi* of amplified (N)	NA (%)	*T. cruzi* of amplified (N)
SAT	5	87.0% (23)	22	100.0% (15)	0	82.6% (23)	27 (30.7)	88.5% (61)
kDNA	1	7.4% (27)	0	32.4% (37)	0	4.3% (23)	1 (1.1)	17.2% (87)
18S	10	11.1% (18)	33	25.0% (4)	17	33.3% (6)	60 (68.2)	17.9% (28)
ME LI	13	26.7% (15)	17	0/20	7	25.0% (16)	37 (42.0)	15.7% (51)
ME LII	10	0/18	37	-	8	0/15	55 (62.5)	0/33
24S	3	0/25	20	0/17	6	0/17	29 (33.0)	0/59

SRN: Santos Reyes Nopala; SCP: Santa Cruz Papalutla; SC: Salina Cruz; specificity (%): # sequences with identity/(no identity + identity); NA: no amplification; SAT: satellite DNA; kDNA: kinetoplast DNA; 18S: small subunit 18S ribosomal; ME: mini-exon; 24S: 24Sα ribosomal.

TABLE IV

Correlation of (A) individual conventional (S1-S4) and rapid (RDT) serological assays with serological (≥ 2 tests) and global (combined serological and molecular) diagnoses, and (B) both end point polymerase chain reaction (epPCR) and quantitative polymerase chain reaction (qPCR) results with global diagnosis, for primary diagnosis group (PDG - above diagonal) and secondary diagnosis group (SDG - below diagonal)

A - Serological assays

		PDG						
		S1	S2	S3	S4	RDT	Dx serology	Dx global
SDG	S1	1	0.807	0.807	0.807	0.807	1.000	0.107
	S2	1.000	1	1.000	1.000	1.000	0.807	0.087
	S3	1.000	1.000	1	1.000	1.000	0.807	0.087
	S4	0.951	0.951	0.951	1	1.000	0.807	0.087
	RDT	0.864	0.864	0.864	0.909	1	**0.807**	0.087
	Dx serology	1.000	1.000	1.000	0.951	**0.864**	1	**0.107**
	Dx global	-0.433	-0.433	-0.433	-0.373	-0.352	**-0.433**	1

B - Molecular assays

		PDG					
		epPCR SAT	epPCR kDNA	epPCR ME	qPCR SAT	qPCR kDNA	Dx global
SDG	epPCR SAT	1	0.375	0.093	0.113	0.224	**0.649**
	epPCR kDNA	0.090	1	-0.093	-0.217	-0.207	0.243
	epPCR ME	-0.152	0.564	1	0.127	-0.214	0.061
	qPCR SAT	0.283	0.358	0.202	1	0.579	**0.478**
	qPCR kDNA	0.251	0.369	0.369	0.663	1	0.564
	Dx global	**0.413**	0.054	0.133	**0.468**	0.251	1

kDNA: kinetoplast DNA; ME: mini-exon; SAT: satellite DNA.

TABLE V

Molecular diagnosis using end point polymerase chain reaction (epPCR) sequences [satellite DNA (SAT), kinetoplast DNA (kDNA), mini-exon (ME) or subunit 18S ribosomal (18S) gene fragments] and quantitative polymerase chain reaction (qPCR) (SAT or kDNA) of *Trypanosoma cruzi* serology-positive (≥ 2 conventional tests) and serology-negative samples [primary diagnosis group (PDG) and secondary diagnosis group (SDG) combined]

	Sequence epPCR							Threshold cycle qPCR						
								N	SAT		kDNA			
T. cruzi serology (N)	N	SAT	kDNA	ME	18S	≥ 2 genes	Global epPCR ≥ 1g		Q+NQ	% NQ	Q+NQ	% NQ	Global qPCR ≥ 1g	*T. cruzi* by both PCR
Negative (57)	43	95.4%	27.9%	9.3%	9.3%	41.9%	75.4%	35	80.0%	7.1%	77.1%	28.6%	61.4%	59.2% (49)
Positive (31)	14	92.9%	21.4%	28.6%	7.1%	42.9%	45.2%	16	87.5%	0.0%	12.5%	100.0%	51.6%	66.7% (18)
Total (88)	57	94.7%	26.3%	14.0%	8.8%	42.1%	64.8%	51	82.4%	5.0%	56.9%	31.3%	58.0%	61.2% (67)

NQ: not quantified positives; Q: quantified positives.

TABLE VI

Satellite DNA (SAT) haplotypes from all sites and according to serology and primary diagnosis group (PDG) or secondary diagnosis group (SDG). Haplotypes in bold are repeated among sites, between diagnostic groups, or serological results

	Haplotypes			
	Previous Dx *Trypanosoma cruzi* (N)	Seropositive (11)	Seronegative (29)	Unique/total haplotypes
SC	PDG (6)	-	**2**, 3, 4, 5, 10, 12	5/6
	SDG (8)	-	1, **2 (2)**, **6**, 7, 9, 11, 12	5/7
SCP	PDG (12)	-	**2**, 14, 15, **16 (3)**, 17, 19, 20, 21, 22, 23	8/10
	SDG (3)	**2**, 13, 18	-	2/3
SRN	PDG (9)	34, 36	**6**, **16**, 24, 25, 26, 27, 29	7/9
	SDG (8)	28, 30, 31, 32, 33, 35	**2**, **16**	6/8
Oaxaca	PDG (27)	2/2	18/21	20/23
	SDG (19)	8/9	5/8	13/17

Oaxaca populations: SC: Salina Cruz; ; SCP: Santa Cruz Papalutla; SRN: Santos Reyes Nopala.

TABLE VII

Satellite DNA (SAT) haplotypes from Oaxaca samples and their multiple *Trypanosoma cruzi* lineage identity (query cover/identity), based on the first 100 matches

SAT haplotype	TcI	TcIII	TcV	TcVI	Matches
H12	99/98[1]	98/96	98/96	96/97	1303
H1	99/99[2]	98/99	98/96	98/96	1299
H2	99/99[1]	97/97	97/97	96/96	1303
H3	100/99[3]	99/97	99/96	99/97	1307
H4	95/95[3]	-	-	-	1265
H5	98/97[3]	98/96	-	98/96	195
H6	99/99[2]	98/98	98/97	98/96	315
H7	99/99[2]	98/97	98/97	98/98	1308
H8	99/99[2]	98/97	98/98	98/98	1308
H9	99/96[1]	-	98/95	98/95	195
H10	99/98[2]	98/97	98/97	96/99	312
H11	99/90[2]	99/88	99/87	-	94
H13	99/99[2]	98/97	98/97	98/98	1303
H14	99/96[2]	98/94	98/94	98/94	1177
H15	99/98[2]	99/96	99/97	99/97	1129
H16	99/100[1]	98/98	98/99	98/98	1308
H17	99/98[2]	98/97	98/97	98/97	1307
H16	99/100[1]	98/98	98/99	98/98	1308
H18	99/94[1]	-	95/94	95/94	1251
H19	99/97[2]	98/97	98/96	98/95	295
H16	99/100[1]	98/98	98/99	98/98	1308
H20	98/98[2]	98/97	98/97	98/97	315
H21	99/99[1]	98/97	98/97	98/98	1303
H22	99/95[2]	99/95	98/94	99/95	291
H23	94/95[2]	94/94	94/94	94/95	194
H30	99/91[3]	99/91	96/90	99/91	291
H31	98/93[2]	99/92	98/92	99/92	7
H24	99/97[1]	98/95	98/96	98/97	293
H32	97/89[3]	92/89	97/87	-	20
H33	99/94[1]	99/91	99/92	99/92	191
H25	99/99[2]	98/99	98/98	98/99	315
H34	99/97[1]	99/95	99/95	99/95	1270
H26	99/97[2]	98/97	98/97	98/97	1293
H35	99/98[2]	99/96	99/95	99/97	1286
H27	99/98[2]	99/96	99/95	99/97	654
H28	99/92[1]	93/93	98/89	99/91	1221
H36	99/96[3]	98/95	98/96	98/96	1294
H29	98/91[3]	-	-	98/95	1225

T. cruzi lineage I (TcI): 1: CARI06; 2: Las Palomas; 3: Silvio X10; *T. cruzi* lineage III (TcIII): strain 3869 and M6241; *T. cruzi* lineage V (TcV): B147 and 115; *T. cruzi* lineage VI (TcVI): CL Brener.

TABLE VIII

Genotype identification using the mini-exon (ME) and 24Sα ribosomal DNA (24S) fragments for all *Trypanosoma cruzi* populations identified using end point polymerase chain reaction (epPCR) (N = 57)

	LI (350) alone	LI + LII	LII (300) alone	NO Amp ME
ME amp (N)	20	21	8	8
ME identity *T. cruzi*	3	5	0	0
Specificity ME amplicons	15.0%	23.8%	0%	0%
24S amp (% amp of ME amp)	17 (85.0%)	16 (76.2%)	8 (100.0%)	5 (62.5%)
Specificity 24S amplicons	0%	0%	0%	0%

ME lineage I (LI); ME lineage II (LII).
